# Posterior Fossa Tumor Rehabilitation: An Up-to-Date Overview

**DOI:** 10.3390/children9060904

**Published:** 2022-06-16

**Authors:** Daniela Pia Rosaria Chieffo, Federica Lino, Valentina Arcangeli, Federica Moriconi, Paolo Frassanito, Luca Massimi, Gianpiero Tamburrini

**Affiliations:** 1 Clinical Psychology Unit, Fondazione Policlinico Universitario Agostino Gemelli IRCCS, 00168 Rome, Italy; federica.lino@policlinicogemelli.it (F.L.); valentina.arca@gmail.com (V.A.); fefes.moriconi@gmail.com (F.M.); 2 Department Women Children and Public Health, Catholic University of Sacred Heart, 00168 Rome, Italy; 3 Pediatric Neurosurgery Unit, Fondazione Policlinico Universitario Agostino Gemelli IRCCS, 00168 Rome, Italy; paolo.frassanito@policlinicogemelli.it (P.F.); luca.massimi@policlinicogemelli.it (L.M.); gianpiero.tamburrini@unicatt.it (G.T.); 4 Department Ageing, Neurosciences Head Neck and Orthopedics Sciences, Catholic University of Sacred Heart, 00168 Rome, Italy

**Keywords:** neurorehabilitation, cognitive remediation in children, tech mediated rehabilitation, posterior fossa tumor, brain tumor

## Abstract

This narrative review highlights the latest achievements in the field of post-surgical rehabilitation of posterior fossa tumors. Studies investigating the effects of cognitive rehabilitation programs have been considered, following a comprehensive literature search in the scientific electronic databases: Pubmed, Scopus, Plos One, and ScienceDirect. This review investigates the effects of cognitive remediation, with specific highlights for single cognitive domains. The results revealed that in spite of the increasing number of children who survive into adulthood, very few studies investigated the effects of rehabilitation programs in this specific population. This study details new, promising therapeutic opportunities for children after brain surgery. More research in this filed is needed to identify the most effective protocols for clinical use.

## 1. Introduction

Pediatric brain tumors are the leading cause of solid tumor mortality in childhood [1].

Tumors of the skull base, such as posterior fossa tumors, were, in the past, associated with a poor prognosis. The evolution of surgical techniques, together with the greater knowledge of the anatomy and phisiology of these diseases and the progress made in diagnostic techniques, now allow treatments that are also curative. 

With the advancement in diagnostic strategies, neurosurgical techniques, and therapies over the past 30 years, an increasing percentage of children with brain tumors survive into adulthood [2]. It is necessary to devote attention to rehabilitation programs that can accompany these children in the developmental arc to gain the best possible quality of life.

## 2. Pediatric Brain Tumor

According to Lehmann and colleagues, the “Brain and other nervous system tumors are of core importance in cancer rehabilitation because of their extremely high rate of disabling sequelae” [3]. According to Fountain and Burke [4], central nervous system tumors are the most common pediatric cancers after hematological malignancies, and the most common pediatric solid organ tumor.

Cognitive deficits are described by Mukand [5] as one out of the three most common complications in rehabilitation for brain tumors. In such a framework, cognitive complications are present in 80% of patients in acute rehabilitation after brain surgery. The other most common complications are represented by focal deficits (78%) and perceptual deficits (53%). 

In the last decade, some reviews have been completed on rehabilitation techniques across specific domains for children with acquired brain injury [6,7]. Some other reviews investigated, more specifically, the functional outcome of different rehabilitation models [8,9].

The present narrative review explores the most diffuse rehabilitation techniques for children with brain tumors. We investigated the use of techniques across the different cognitive domains. In particular, our attention was oriented to explore the rehabilitation techniques dedicated to children with a specific condition among the others in the wide field of brain tumors: posterior fossa tumors, one of the most common brain tumor location in children younger than 10 years old [10,11]. 

## 3. Methods 

The present narrative review is based on research conducted on Pubmed, PsycINFO, and Scopus databases, all accessed by February 2022. Searches were not filtered by year. We incorporated reviews, meta-analyses, and systematic reports. Single-case studies were excluded. Non-English studies were not considered as well. Our literature search was based on the following search terms: “posterior fossa brain tumor in children” or “posterior fossa syndrome” or “cerebellar mutism syndrome”. Afterwards, we combined the abovementioned search terms with “cognitive rehabilitation” or “cognitive training” or “brain functons rehabilitation”. We considered a total of 300 papers. 

## 4. Posterior Fossa Tumor Rehabilitation

In the last decades, as survival rates in brain tumors have risen [12,13], it has become imperative to investigate which kind of interventions are the ones that fit best with children needs. Cognitive remediation/rehabilitation therapy is based on the principles of neural plasticity of the brain. It is a type of rehabilitation asking the patient to train with cognitive exercises to booster several cognitive functions. Historically, greater efforts have been directed to cognitive rehabilitation in adulthood and in particular, in patients with other types of acquired brain injuries (i.e., stroke and traumatic brain injury). Cognitive rehabilitation interventions can be helpful for patients with tumors too. Rehabilitation exploits the principles of plasticity and functional compensation, possible after a stroke, as well as after brain surgery. Cognitive intervention to implement a remodeling of functional networks, combined with pharmacological treatment, can improve the residual functional capacity [14]. 

Despite the growing interest in rehabilitation after pediatric brain tumor surgery, the literature of proven effective interventions is still poor.

In 2015, Fountain and Burke [4] investigated the evidence base for multidisciplinary rehabilitation in the children and adolescent population with brain tumors. In their review, they found that no study considered a specific tumor type, and all the studies they identified were single-center-based studies. They concluded that a multi-centre study with a cost-effective strategy, standardized procedures, and long-term analysis is needed.

A dissertation across methods used insingle cognitive domains rehabilitation will follow. 

## 5. Executive Functions

Pediatric brain tumor is known to impact attention and executive functions, such as working memory (WM; temporary storage and manipulation of information), as well as children’s metacognition, which involves abilities such as evaluating own performance and estimating own ability to learn. [15]. A deficit involving the abovementioned abilities together with a lack in the speed processing can lead to intellectual decline in children [16].

Deficits of executive functions are present in many cerebellar pathologies. In the last decade, studies are investigating more in-depth connectivity models of brain functioning. In this framework, the concept of a processing network for executive functions has been developed. In the words of Gazzaniga, “Executive functions do not reside in a single structure but result from the interplay of diverse cortical and subcortical neural systems” [17]. Histological studies have confirmed a connection between cerebellar areas and frontal and prefrontal areas [18,19]. In children with posterior cranial fossa tumor, there could be an executive function damage. 

As a contributing factor, the presence of a posterior fossa tumor may act similarly to a local trauma, favoring the appearence already at diagnosis of neuropsychological deficits, as well as favoring their persistence/worsening after surgery. In this context, M.J. Hylin and colleagues have suggested that endoplasmic reticulum (ER) stress may be the responsible contributing factor to injury expansion, leading to behavioral deficits; they documented this occurrence in their experimental rat model. Binding immunoglobulin protein (BiP) and C/EBP homologous protein (CHOP) were measured 4 h after local injury in the ipsilateral pericontusion cortex. Hypoxia-inducible factor (HIF)-1α was measured at 48 h, and tau kinase measured at 1 week and 30 days. At 4 h following injury, BiP and CHOP (markers of ER stress) were significantly elevated in rats exposed to TBI. HIF-1α was significantly upregulated 48 h following TBI showing delayed hypoxia. The early ER stress activation was additionally associated with the activation of a known tau kinase, glycogen synthase kinase-3β (GSK-3β), by 1 week. Tau oligomers measured by R23 were significantly increased by 30 days following TBI. The biochemical changes following TBI were associated with increased impulsive-like or anti-anxiety behavior measured with the elevated plus maze, deficits in short-term memory measured with novel object recognition and deficits in spatial memory measured with the Morris water maze in juvenile rats exposed to TBI [20].

Cognitive remediation programs could be distinguished in two main approches: the face-to-face remediation approach and the computerized approach [21]. 

To the best of our knowledge, the most comprehensive and recent face-to-face remediation research program [22] was a phase 3 trial of a multidimensional program which showed a clear efficacy in improving attention and metacognitive skills. The study targeted three areas: hierarchically graded massed practice, strategy acquisition, and cognitive-behavioral therapy. One-hundred and sixty-one survivors of childhood malignancy aged between 6 and 17 years old participated in the study. Limits for this cognitive remediation modality stand in the time effort to be dedicated by the families of these children. All participants underwent a neuropsicological testing baseline. Participants were seen for a total of up to 22 h weekly sessions over a time of 4 or 5 months. Neuropsychological battery investigated attentional functions, memory, new learning, and academic achievements. Researchers also collected parents’ and teachers’ reports on executive abilities and quality of life. The primary outcome measures considered were: Standardized academic achievement tests, Wide-Range Achievement Test—Third Edition, Calculation and Applied Problems (Woodcock–Johnson Tests of Achievement—Revised), Reading Comprehension (Peabody Individual Achievement Test—Revised), Arithmetic (WISC–III), Digit Span (WISC–III), Sentence Memory (Wide-Range Achievement Test of Memory and Learning (WRAML)), Stories (Children’s Memory Scale), Rey Auditory Verbal Learning Test (Trial 1 (RAVLT)), Digits Backward (WISC–III), Stroop Color–Word Test (Trial 3), Trail Making Test B, Brief Test of Attention, Stories (Delayed Recall), Rey–Osterrieth Complex Figure Test (Delayed Recall), RAVLT (Delayed Recall of Trial 1), and CPT–II. This study demonstrated a statistically significant improvement in academic performance, metacognitive strategies, and attention (by parents’ reports).

Patel and colleagues investigatedthrough a pilot study, the use of a 15 sessions training aiming the rehabilitation of executive functions and problem-solving and oriented to cognitive domains of attention [23]. Despite 70% of the families completed the course, the researchers found poor compliance with the treatment; more studies are needed to confirm the efficacy of this protocol.

It is well known that the use of technology in everyday life is increasing. At the same time, technological tools for cognitive rehabilitation in children with brain tumors are rising. Traditional in-person cognitive rehabilitation methods may be unavailable and/or impractical for many of these children in terms of time resources needed and health status.

Cogmed has been proven, to the time being, to be the best computerized cognitive training, with demonstrated efficacy for acquired attention and executive function disorders [24]. In a randomized controlled trial [25], participants aged between the age of 8 and 16 years underwent 25 Cogmed training sessions over 5 to 9 weeks. Training sessions consisted of visual–spatial and verbal WM exercises proposed as games. Each session duration was 30 to 45 min. Exercise difficulty was adjusted based on performance. A baseline evaluation was collected, as well as a post-treatment evaluation after 10 weeks from baseline. Cognitive tests to evaluate training gains were an abbreviated IQ (obtained from WASI vocabulary and matrix reasoning tests), WISC verbal span, digit span and number letter sequencing (to assess working memory), The Conners’ Continouus Performance Tests (to assess sustained attention), and The Behaviour Rating Inventory of Executive Functions (wich is a parent questionnaire on executive functions). A neuroradiological evaluation through fMRI [26] was given to investigate the neural correlates of WM performance and the changes after Cogmed training (during fMRI participants completed a WM task). The study results showed computerized cognitive training through Cogmed tool as an efficacious, portable, motivating, and less time-intensive alternative to traditional cognitive interventions.

Hardy, Willard, and Bonner (2011) explored the effectiveness of a computerized home intervention through a pilot study. The duration was estimated in 12 weeks. Nine children with previous brain tumors, of which, six had a previous posterior fossa tumor, completed the program, entitled “Captain’s Log”. Researchers found improvement in scores from baseline to 3 months after surgery; parent report measures were used, which also demonstrated improvement in attention [27]. The most interesting detail of this program was that Hardy and colleagues had the pioneering initiative to also include outcome measures of executive functions other than those related to attention [16].

In 2018, Van Der Linden and colleagues further developed the logic of computerized rehabilitation by implementing the first evidence-based program of telerehabilitation to specifically accompany the postoperative course of adult patients with brain tumor through the use of an iPad-based App called Re-Mind [28]. Looking at the new technologies, Yang and colleagues investigated the use of VR tools in treating postoperative brain tumor cognitive dysfunctions. They found that VR tools can be effective when associated to computer-based cognitive rehabilitation [29].

Further studies are needed to better understand the potential of this kind of tool in pediatric age. 

## 6. Visuo Spatial Skills

Pediatric posterior fossa tumors have been associated with neuropsychological sequelae in terms of visuospatial organization [30]. Children with cerebellar damage may display deficits in complex visuospatial processes such as mental rotation, visuospatial organization, and planning [31]. Starowicz and colleagues specifically explored the link between the side of cerebellar lesions and the level of visuospatial impairments. Visuospatial deficits have been observed in several previously published studies considering adults with cerebellar lesions, whereas others have been centered on the pediatric population. Depending on the side of cerebellar lesion, a specific cognitive-damage profile can be depicted. [32,33,34]. However, there is a lack of randomized controlled studies specifically dedicated to visuospatial function rehabilitation in children treated for a brain tumors, and no studies are present for specific tumor locations. 

Recently, Corti and colleagues [35] have shown the efficacy of remote computerized brain training in children with acquired brain injury. Even if children with brain tumors were excluded in this study, the paper is interesting for its results. The authors administred an 8-weeks training program through home-based computer training (Lumosity). This tool is a web-based platform providing game-like exercises. The performance in visual-spatial working-memory improved more after the training than after the waiting-list period, showing efficacy and near-transfer effects. The abovementioned study proposed five games, focusing on different cognitive domains, and relied on visual-spatial competence. Games focused on: detecting the orientation of a stimulus in space (Disillusion, Lost in Migration), matching together (Disillusion) or recognizing figures different in shape and colours (Tidal Treasure, Speed Match), solving arithmetic operations presented in drops that moved vertically on the computer screen (Raindrops), maintaing in working memory the shapes and colors of visual stimuli (Tidal Treasure, Speed Match). The primary outcome in this study was the Corsi block-tapping test. In the Section 10, the authors suggest that “the best cognitive benefts in pediatric acquired brain injury are achieved by the intensive stimulation of the same cognitive function”.

## 7. Language 

In posterior fossa syndrome, language is the primarily-affected cognitive function in the post-operative period. Postoperative cerebellar mutism syndrome (pCMS) may develop after posterior fossa tumor surgery. It indicates mutism and speech deficits [36], occurring in about the 25% of the cases [37]. pCMS is described as an upcoming mutism (usually, 48 h after surgery), followed by motor speech deficits, motor deficits, and emotional and behavioral symptoms. Concerning language, early after surgery, a transient mutism may occur, which may last days or months (until six), usually followed by a period of dysarthria. During the mutism phase, verbal sounds are not produced, but other sounds, such as crying or laughter, have been frequently described in clinical reports, and when this phase ends, dysarthria and dysphagia are often found. The condition of mutism is, in most cases, transient, but as previously stated, the efforts for a complete recovery can be prolonged, and a complete return to the premorbid state may not occur. Somehow, the length of the mutism recovery period seems to be linked to a worse recovery prognosis [38]. Motor/speech deficits after mutism have been estimated as occurring in 98.8% of the total children with pCMS [38,39]. The clinical picture may be accompanied by cerebellar motor syndrome.

Although a series of cases have been described in the adult population, pCMS is referred to as a pediatric condition. This condition is present in a variable percentage, described between 8 and 31% [39]. It is also much more common for children to have posterior fossa syndrome, as the cerebellum is one of the most frequent locations in pediatric brain tumors under 10 years of age [40]. Currently, there is no validated efficacy protocol for pCMS rehabilitation [41].

It is evident that the rehabilitation plan of the syndrome requires a multidisciplinary intervention (since various types of disorders are present) and long-distance planning, in light of the duration of the diturbance which has been described as variable between 1 day and 6 months [39] Of course, execptions in duration have been described in literature. 

Children with pCMS have a worse prognosis over time, showing greater intellectual, cognitive and behavioral difficulties. Robertson and colleagues also devised a pCMS severity scale that takes into account the time to onset of symptoms after surgery and the duration of symptoms, attributing severity on the basis of the number and duration of symptoms [42]. 

The clinical picture in the developmental age is complicated by the fact that it concerns patients who are developing cognitive and motor functions [43], and whose acquisition could be delayed compared to the typical development phase. For this reason, it is desirable to develop programs aimed at the longitudinal observation of patients in rehabilitation. As previously discussed, Paquier [41] describes two types of rehabilitative approaches: those face-to-face-oriented to single cognitive domains and those characterized by the use of computers that also enable an home setting. From the face-to-face side, the rehabilitation program that has been proved most effective to date is a phase 3 study with a multidimensional approach. The program is oriented to: massive practice, strategies’ acquisition, and cognitive behavioral therapy. As it can be guessed, such an approach has as its weak point, the difficulty of families in carrying out such a demanding rehabilitation program. From the home setting side, a computerized program called Cogmed has proven to be effective, in particular, in working memory and visuospatial functions stimulation with encouraging proven benefits [25].

Patients usually need 6 months to recover from pCMS. At a later time, if recovery has not been completed, most children suffer from residual motor speech deficits in long-term follow-ups. [44]. In the past, most authors looked optimistically at pCMS recovery [45] but most recent studies have confirmed a worse than expected prognosis for pCMS, seeing that a large proportion of children have been found suffering from related sequelae years after surgery [46,47]. 

There is no international agreement or estabilished treatment for children with pCMS. Of course, a multidisciplinary, integrated approach is needed, which should combine earliness and regularity over time, to follow the upcoming needs of a developing brain [41]. A speech assessment is needed as a baseline, which subsequently stands as the basis for carrying out follow-up assessments. This type of work is necessary in order to outline specific rehabilitation protocols through evidence-based procedures. In their study, Di Rocco and colleagues [46] confirmed that pre-operative language impairment has been shown as a strong risk factor for pCMS. Baseline deficits can be characterized as reduced spontaneous language, decreased mean length of utterance, difficulties in word-finding abilities, and in phonological-driven word production tasks. In addition, the brainstem invasion has been demonstrated as a prognostic factor in the same study.

Paquier [41] identifies five steps for motor speech rehabilitation in children:

A pre-surgical speech-evaluation as a baseline (which could identify predictors and risk factors);

A post-surgical speech-evaluation which should define the real deficits; 

The determination of the specific rehabilitation that best fits with patient needs;

Follow-up evaluations to monitor symptoms over time;

A prospectic evaluation to determine risks that language deficits could generate on children as a function of quality of life is needed: children’s needs, in terms of rehabilitation, are influenced by the effects of a potential language deficit on school career, and later, on job-seeking.

## 8. Ataxia and Motor Problems

It is sometimes possible to observe motor difficulties, hypotonia, walking difficulties, postural deficits, or ataxia in children with pCMS [48].

Ataxia is present, according to Wilne and colleagues [49], in 60% of children with pCMS. Specific tumor locations are predictors of a long-term ataxia [50]. 

Motor therapy is often included in a multidisciplinary rehabilitation approach in pediatric patients. Though there is no scientific evidence for the efficacy of this approach, there is an indication in the literature for conventional physical therapy for children with ataxia in the general context of acquired brain injury. Sabel and colleagues [51] investigated the efficacy of a home-based video game training for balance training in children after brain tumors. The training was based on an active video game training using an “off the shelf” tool: the Nintendo Wii. On the other side, the literature suggests that children with balance problems recovering from brain tumors could benefit from treadmill training [52].

Extensive literature documents the use of technologies such as VR tools in motor rehabilitation following acquired brain injury in adult patients. Some studies also highlight the role played by the combination of the use of robotics and VR tools in the effectiveness of rehabilitation treatments. More studies are needed to confirm the results in children [53]. Barbarulo and colleagues have recently observed in their study on multiple sclerosis that the combination of cognitive and motor rehabilitation has “mutual improvement effect on motor outcome compared to their independent administration” [54]. This could be linked, according to Manuli and colleagues, to an improved adaptive neuroplasticity that would find justification in the involvement of shared neural circuits (cortical-subcortical), most of which would refer to the mirror neuron system. The abovementioned network is not only involved in motor movement, but also in movement planning. Movement planning needs the attention of more primarily executive functions that confirm the grip relationship between movement and cognition. Considering this framework, VR could bring better cognitive–motor–behavioral outcomes because it can combine physical exercise with cognitive excercise, and both sensorymotor function and cognitive inputs would be given in the virtual environment. More research in this field is needed to identify evidence-based protocols to be applied for children’s rehabilitation.

## 9. Behavioral and Emotional Disturbances

Behavioral symptoms may occur in the pre- and post-surgical period following resection of a tumor in the posterior cranial fossa. 

A change in behavior as a reduction of feeding and increased fuzziness have been reported, for example, to mask, particularly in smaller children, signs of increased intracranial pressure, delaying the diagnosis [55].

Due to their common persistence after surgery, it is necessary to treat behavioral symptoms that can be very disabling in order to make the child an active part of the rehabilitation. In 2017, Lanier and colleagues [56] summarized in a review the near- and long-term psychosocial and psychiatric implications of emotional and behavioral symptoms. They also depicted a useful and comprehensive overview for clinicians, providing clinical examples of the presentation, management, and lasting implications of posterior fossa syndrome.

Emotional and behavioral symptoms may accompany the mutism phase when posterior fossa syndrome is present. From a behavioral perspective, marked apathy and lack of interest for the environment with decreased responsiveness to surrounding stimuli may occur. Physical agitation, restlessness, poverty of spontaneous movements, and withdrawal may also be present. The emotional symptoms most commonly described in the literature are irritability, dysphoria, lability, tearfulness, and inconsolability. These manifestations cannot uniquely be linked to the frustration experienced by the child because of speech deficits. Very often, in fact, emotional symptoms persist even when speech deficits are improved.

Children after posterior fossa tumor surgery may show emotional or behavioral symptoms even years after surgery [57,58,59,60,61].

The manifestation of behavioral problems is directly related to: exact location of the tumor, treatments such as radiotherapy, onset of complications, or other medical conditions [62,63].

The most damaged functions in this type of patients seem to be those related to more frontal neuroanatomic areas, with consequent behavioral outcomes (predominantly dorsolateral frontal expression).

Di Rocco and colleagues [46] studied the correlation between pre-operative and post-operative disorders. They investigated the presence of speech disturbances and preoperative behavioral disturbances (sleep disturbances, hyperactivity, and somatic disturbances). What was found was that all children who developed postoperative cerebellar mutism (i.e., 20.6%) had preoperative disturbances. 

The recovery, as it can be easily inferred, requires targeted behavioral therapy, physical therapy, occupational therapy, and speech therapy.

Given that a persistent motor speech disorder is often present [47], it would be useful, as Hocking highlighted in his study [64], to consider a component of social reintegration as part of the rehabilitation programs for supporting social skills for these children.

## 10. Discussion

Today, it is possible for a child who has undergone brain tumor surgery to look to the future with greater confidence, as there have been many advances in the field of surgery, treatment, and rehabilitation. The developmental age presents numerous challenges in terms of “rehabilitation after brain surgery”, since it is a sensitive window in which some functions are acquired for the first time. A delay in the acquisition of certain skills can lead to cognitive, motor, and psycho-emotional damage. A great effort is required to be able to take in charge patients at a developmental age by looking at the rehabilitative intervention within a multi-disciplinary, multisystem, and multi-sensory approach, bearing in mind that measuring both the short- and long-term effects of brain tumors in a developmental age is the first step towards improving their clinical course [65].

Neurorehabilitation has, in recent decades, moved from a “bottom-up” to a “top-down” approach. This change of perspective has been possible thanks to technological devices developed for motor and cognitive rehabilitation. This evolution has made possible new therapeutic approaches targeting brain stimulation in a more direct way to elicit plasticity-mediated learning [66].

We now know that timely management is directly linked to the outcome of rehabilitation, in conjunction with factors such as age, tumor site, and post-surgical treatment. It has salso been shown that some pre-operative features can predict postoperative symptoms [48]. It is therefore essential to pay attention to these aspects, and implement tailored rehabilitation protocols taking into account the evolutionary needs and stages of development. To address cognitive disorders following posterior fossa tumor surgery, it is desirable to rely on multidisciplinary protocols that boost the use of computerized technologies, making the rehabilitation moment motivating for the child.

Today, new technologies have opened new windows of possibilities on rehabilitation following brain tumor in the pediatric age. The use of computers and digital tools allow clinicians to administer more patient-oriented and tailored treatments. Computer-based interventions also make possible rehabilitation at the patient’s home, with indisputable logistical and psycho-emotional advantages. The use of technologies such as brain computer interfaces, robotics, virtual reality, and augmented reality tools currently adopted in the treatment of cognitive disorders in adult patients and children [67,68,69,70,71,72,73] could also lead to indiscuss advantages in terms of motivation in posterior fossa tumor post-surgery rehabilitation.

Further studies are needed to verify the efficacy of protocols that have the necessary multidisciplinary characteristics in the post-operative course of children with posterior cranial fossa tumors.
